# Prevalence, Incidence and Etiology of Hyponatremia in Elderly Patients with Fragility Fractures

**DOI:** 10.1371/journal.pone.0088272

**Published:** 2014-02-05

**Authors:** Kirsten Cumming, Graeme E. Hoyle, James D. Hutchison, Roy L. Soiza

**Affiliations:** 1 Acute Geriatric Medicine, Aberdeen Royal Infirmary, Aberdeen, United Kingdom; 2 Surgery and Orthopaedics, University of Aberdeen Medical School, Aberdeen, United Kingdom; University of Glasgow, United Kingdom

## Abstract

**Introduction:**

Hyponatremia (serum sodium<135 mMol/L) is the commonest electrolyte imbalance encountered in clinical practice. It is associated with multiple poor clinical outcomes including increased mortality, longer hospital stay, falls and fractures. Prevalence is higher in frail patient groups, and elderly patients with fragility fractures (EPFF) are particularly susceptible. Euvolemic hyponatremia due to syndrome of inappropriate anti-diuretic hormone (SIADH) is widely assumed to be the commonest cause. However, little is known about the epidemiology and etiology of hyponatremia in EPFF. This study established prevalence, incidence and etiology of hyponatremia in EPFF.

**Methods:**

Prospective observational study of consenting adults aged ≥65 years admitted with a fragility fracture to a university hospital between 7th January and 4th April 2013. Prevalence of hyponatremia on admission and incidence of cases developing in hospital were reported. Etiology of cases of hyponatremia was determined by consensus of an expert panel using pre-specified data collected daily.

**Results:**

127/212 (60%) EPFF were recruited (mean age 79 yrs, 78% female). Two participants withdrew mid-study. Of those not recruited, 66 had incapacity to consent and 19 refused participation. Point prevalence of hyponatremia on admission was 13.4% and a further 12.6% developed hyponatremia during admission. Hypovolemic hyponatremia was predominant (70%). 73% of cases were multi-factorial in etiology. The commonest potentially causative factors in cases of hyponatremia were thiazide diuretics (76%), dehydration (70%), proton pump inhibitors (70%), SIADH (27%) and mirtazapine (15%).

**Conclusion:**

Hyponatremia is highly prevalent in EPFF, seen in 26% of cases. Dehydration and prescription of thiazide diuretics and proton pump inhibitors were the commonest potentially causative factors, not SIADH.

## Introduction

**Figure 1:**
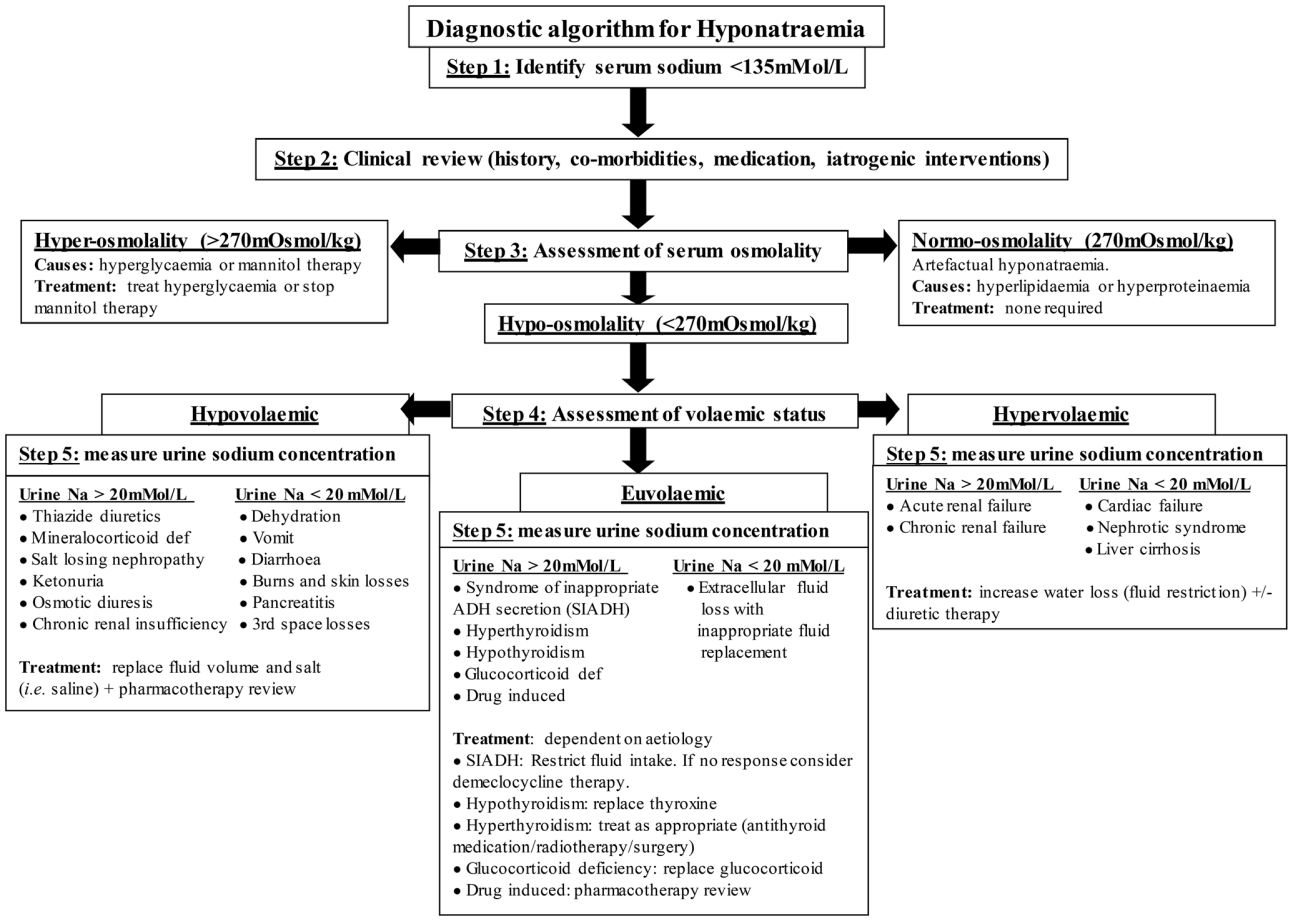
Hyponatremia diagnostic algorithm. Adapted from Soiza *et al.* [1]

Hyponatremia, serum sodium < 135 mMol/L, is the commonest electrolyte imbalance encountered in clinical practice. It is associated with multiple poor clinical outcomes including falls, fractures, increased length of hospital stay, institutionalisation and mortality [1]. Prevalence is known to increase in frail patient groups, such as elderly, hospitalised, peri-operative patients with a fracture. Elderly patients with fragility fractures (EPFF) have increased risk of hyponatremia as a result of degenerate physiology, multiple co-morbidities, polypharmacy, increased risk of dehydration due to hospitalisation and peri-operative fluid restriction, and homeostatic stress from fracture and subsequent surgical interventions [2]–[6]. eThey are also at higher risk of complications, making this group of special clinical importance. Hyponatremia itself may be responsible for the fracture [7]. Reports of the prevalence of hyponatremia at admission in EPFF vary widely between 2.8%–26.5%, while 2.6–5.5% develop hyponatremia in the post-operative period [3]–[5], [8], [9]. Hyponatremia occurs due to disruption of sodium and water homeostasis, normally maintained by complex multi-system physiological mechanisms [1]. Consequently, there are numerous potential underlying causes of hyponatremia, spanning a broad spectrum of diseases, pharmacotherapy and pathophysiological variants each with different treatment requirements (see Figure 1) [2].

When very acute or severe, hyponatremia may present with neurological symptoms which can result in serious complications *e.g.* hyponatremic encephalopathy, non-cardiogenic pulmonary oedema, seizures, coma, death [2], [10]–[13]. However, 75–80% of cases of hyponatremia are mild and chronic (*i.e.* serum sodium 130–134 mMol/L, occurring over >24 hours) and typically devoid of obvious neurological symptoms [1], [2], [14]. As a result, chronic mild hyponatremia is frequently considered asymptomatic despite being strongly associated with major geriatric conditions and multi-organ pathological changes. These include abnormal gait patterns, falls, fractures, cognitive impairment, bone demineralisation, longer hospital stay, institutionalisation and increased mortality [2], [5], [6], [9], [12], [15]–[19]. Despite this, older people may be at lower risk of hyponatremic encephalopathy and subsequent complications of acute severe hyponatremia [20], where female gender, hypoxia and liver dyfunction are associated with poorer prognosis [21]. eWhether hyponatremia is an independent predictor of patient outcomes or a marker of disease severity is controversial [2]. Nevertheless, it is very treatable, so its association with multiple poor clinical outcomes is important.

**Figure 2:**
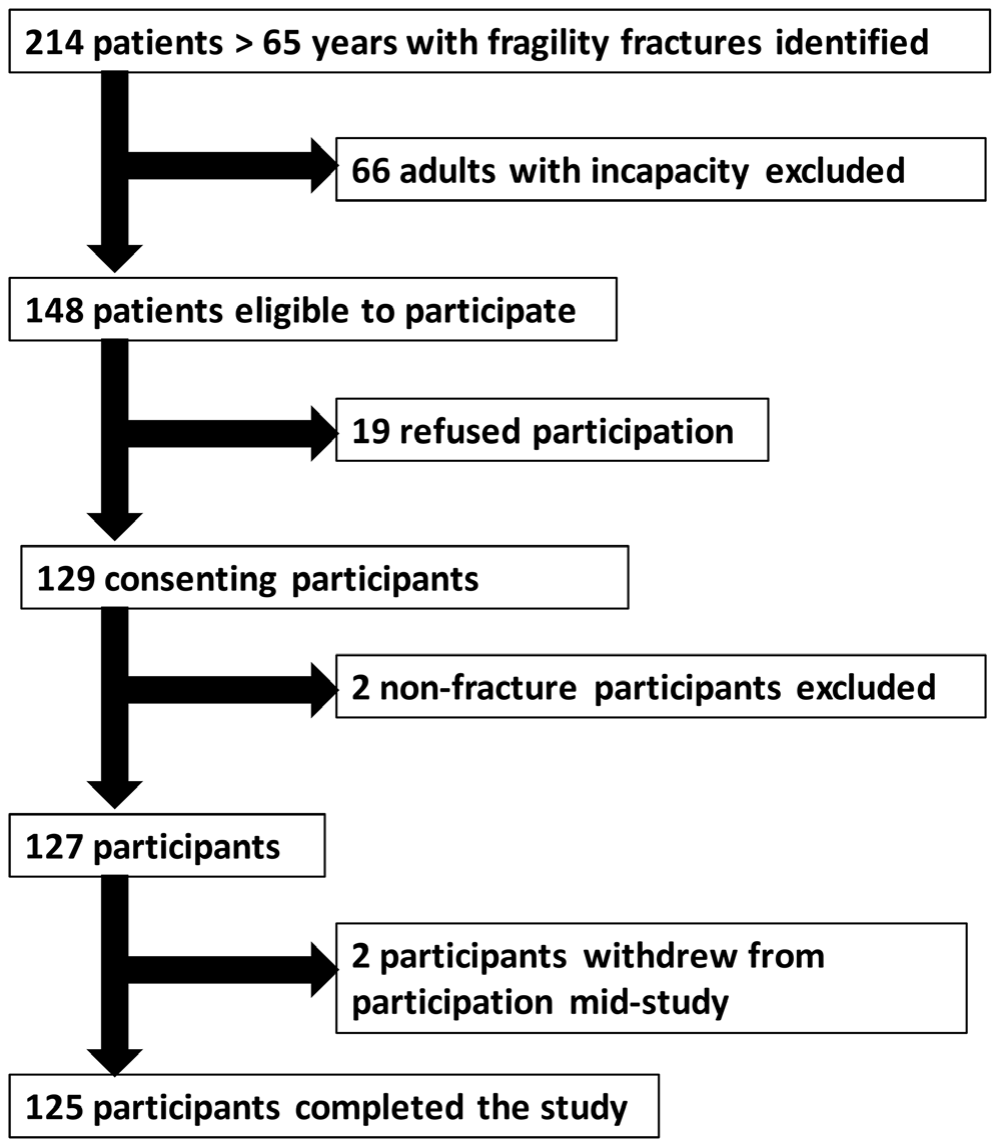
Participant recruitment flow chart.

Clinical management of hyponatremia is based on diagnosing and treating the underlying cause(s) and restoring salt and water balance (see Figure 2) [1]. However, accurate determination of etiology of hyponatremia is notoriously challenging [22]. The best available method to elucidate causes of hyponatremia is by expert panel consensus. Diagnosis depends crucially on accurate assessment of volemic status which is difficult to determine with certainty, especially in older individuals for whom there is currently no reliable biomarker of hydration [2], [20]. Euvolemic hyponatremia due to the syndrome of inappropriate anti-diuretic hormone (SIADH) is widely assumed to be the commonest cause [17]. However, SIADH may be over-diagnosed as a result of diagnostic difficulties, particularly in dehydrated older people [2], [23]. Subsequently, patients may be subject to serious clinical consequences as the management of SIADH (fluid restriction) is the exact opposite of the management of dehydration (vigorous fluid replacement). ee

SIADH is a reportedly common cause of hyponatremia in EPFF along with excess hypotonic fluid, diuretic and antidepressant therapies [3], [5], [8]–[10], but the process of attribution of causation of hyponatremia in these studies did not include expert review or any quality assurance process. Our aim was to undertake the most detailed study to date of the etiology of hyponatremia in EPFF and report its prevalence and incidence.

## Materials and Methods

We carried out a prospective observational study of all adults aged 65 years or over admitted with a fragility fracture to a university teaching hospital from 7^th^ January – 4^th^ April 2013. Fragility fractures were defined as those occurring either without trauma or due to low energy trauma, equivalent to a fall from standing height or less than one metre. Anonymous baseline data (age, sex, fracture site and admission serum sodium) of all identified EPFF were recorded. Adults with incapacity were excluded from recruitment. Capacity to consent was assessed by the attending clinical team in accordance with the Adults with Incapacity (Scotland) Act 2000. Participants were recruited from acute orthopaedic trauma wards and a geriatric assessment unit. Clinical data were collected daily until discharge, from patient interview, medical and nursing notes, observation and fluid balance charts and laboratory computer systems by a single investigator (KC). Medication data were obtained by reconciling patient and/or carer histories, and primary and secondary care records. Also, clinical examination of volemic status was performed daily by the investigator. This involved measurement of skin turgor, capillary refill time, mouth moistness, axillary moistness, jugular venous distension, peripheral oedema and overall impression (signs selected based on previous research recommendations) [22]. Examination was carried out by one investigator to increase reliability of findings, maximise consistency and exclude effects of inter-observer variability. An initial two week training period of examination of volemic status, under supervision of the principal investigator (RLS), was undertaken to help ensure quality and consistency.

Cases of hyponatremia were defined as any serum sodium measurement <135 mmol/L. An expert panel, consisting of two consultant geriatricians with special interest in hyponatremia (RLS and GEH) and one consultant orthopaedic surgeon (JDH) reviewed and determined etiology of each case of hyponatremia. The panel did not examine patients themselves but instead retrospectively reviewed each case of hyponatremia relying on the detailed daily prospectively-collected data and clinical examination findings provided by the investigator. This included all clinical information required to determine underlying cause(s) of hyponatremia - history, medications, detailed daily examination, fluid intake and output charts and laboratory results. Collectively, the expert panel used a diagnostic algorithm (Figure 1) to determine underlying cause(s) of hyponatremia.

Ethical approval was obtained from the Scotland A Research Ethics Committee (Ref 12/SS/0209). The original protocol submitted to the Committee had provision for the inclusion of adults with incapacity to consent but the Committee's approval was dependent on their exclusion. The Committee's recommendations were fully incorporated into the final study design and final approval was obtained from the Committee Scientific Advisor.

The prevalence of hyponatremia upon admission and the incidence of cases developing in hospital were calculated. For incident cases, we recorded whether the hyponatremia was pre- or post-operative. The proportion of participants with known hyponatremia prior to their fracture was calculated by obtaining the last available serum sodium for each patient prior to their admission to hospital. The laboratory is the only public health service laboratory covering the study population so it was unlikely that patients had more recent investigations elsewhere. Prevalence of hyponatremia at discharge was calculated according to the last available serum sodium measurement prior to discharge.

Independent-T tests or Mann-Whitney U tests were used as appropriate. Pearson chi-squared and Fisher's exact tests were calculated to compare proportions across groups. Statistical Package for Social Sciences (SPSS) version 20.0 was used to perform all analyses. Statistical significance was assumed where p<0.05.

## Results

### Recruitment, participant information and data collection

There were 214 EPFF identified and 127 were recruited into the study (see Figure 2). Those not recruited had incapacity to consent (N = 66), declined participation (N = 19), or agreed but were later excluded when the original diagnosis of fragility fracture was excluded (N = 2). There were no statistically significant differences between participants and those who were eligible but declined participation (see Table 1). However, compared to participants, adults with incapacity were on average seven years older (p<0.001) and had different proportions of fractures site, with hip fractures composing n = 47 (71.2%) of fractures compared to n = 67 (52.8%) in participants (p = 0.017). The characteristics and outcomes of the 127 participants are detailed in Tables 2 and 3, categorised according to natremic state.

**Table 1:** Subgroup analysis of baseline data.

	Participants	Non-participants	P value*	Adults with incapacity	P value**
**Number of individuals (N)**	127	19		66	
**Age (years)**					
Mean (± SD)	79 (±8)	77 (±9)	0.438	86 (±7)	<0.001
Range	65–98	65–93		66–101	
**Female sex, % (N)**	78.0 (99)	89.5 (17)	0.365	69.7 (46)	0.223
**Fracture site, % (N)**			0.676		0.017
Hip	52.0 (66)	36.8 (7)		69.7 (46)	
Hip & upper limb	0.8 (1)	0		1.5 (1)	
Other lower limb	22.0 (28)	31.6 (6)		7.6 (5)	
Other lower & upper limb	0.8 (1)	0		0	
Upper limb	19.7 (25)	26.3 (5)		10.6 (7)	
Pelvic and upper limb	0	0		1.5 (1)	
Pelvic	3.1 (4)	5.3 (1)		6.1 (4)	
Vertebrae	1.6 (2)	0		3.3 (2)	
**Admission serum sodium (mMol/L)**					
Mean (± SD)	138 (±4)	138 (±5)	0.556	139 (±4)	0.172
Range	123–146	128–143		124–147	
**Prevalence of hyponatremia on admission, % (N)**	13.4 (17)	26.3 (5)	0.16	12.1 (8)	0.827

*participants v non-participants.

**participants v adults with incapacity.

**Table 2:** Participant characteristics.

Characteristic	Normonatremic participants	Hyponatremic participants	P value
**Number (N)**	94	33	
**Age (years)**			
Mean (± SD)	78 (±8)	81 (±7)	0.083
Range	65–97	69–98	
**Female sex, % (N)**	76.6 (72)	81.8 (27)	0.078
**Previous residence, % (N)**			
Own home	85.1 (80)	78.8 (26)	0.390
Sheltered housing	14.9 (14)	12.1 (4)	
Care home	0	6.1 (2)	
Care of the elderly ward	0	3.0 (1)	
**Number of co-morbidities**			
Mean (± SD)	4 (±2)	5 (±3)	0.222
Range	0–9	0–11	
**Number of co-morbidities that cause hyponatremia (N)**			
Mean (± SD)	1 (±1)	1 (±1)	0.855
Range	0–3	0–4	
**% of participants with at least one co-morbidity that causes hyponatremia (N)**	60.6 (57)	54.5 (18)	0.681
**Number of medications (N)**			
Mean (± SD)	5 (±4)	6 (±3)	0.294
Range	0–15	0–15	
**% of participants on at least one medication that causes hyponatremia (N)**	64.9 (61)	72.7 (24)	0.52
**Fracture site, % (N)**			0.115
Hip	50.0 (47)	57.6 (19)	
Hip and upper limb	1.1 (1)	0	
Other lower limb	24.5 (23)	15.2 (5)	
Other lower limb and upper limb	0	3.0 (1)	
Upper limb	21.3 (20)	15.2 (5)	
Pelvic	3.3 (3)	3.0 (1)	
Vertebrae	0	6.1 (2)	
**Time from fracture to hospital admission (days)**			
Median	0	0	0.581
Interquartile range	0–1	0–1.5	
**Serum sodium prior to admission (mMol/L)**			
Mean (± SD)	140 (±2)	136 (±5)	<0.001
Range	133–145	127–144	
**Serum sodium on admission (mMol/L)**			
Mean (± SD)	139 (±2)	134 (±4)	<0.001
Range	135–146	127–144	
**Prevalence of hyponatremia, % (N)**			
Prior to admission*	3.2 (3)	30.3 (10)	<0.001
On admission	0	51.5 (17)	<0.001

*According to last serum sodium available prior to admission.

**Table 3:** Outcomes in patients with and without hyponatremia.

Characteristic	Normonatremic participants	Hyponatremic participants	P value
**Number (N)**	94	33	
**Surgical management, % (N)**	76.6 (72)	81.8 (27)	0.631
**Time to surgery from admission (days)**			
Mean (± SD)	1.8 (±1.7)	3.0 (±3.0)	0.014
Range	0–7	0–10	
**Patient deaths, % (N)**	2.1 (2)	3.0 (1)	1
**Barthel index at discharge**			
Median	60	57.5	0.918
Interquartile range	45–75	48.75–72.5	
**Decline in Barthel index from admission to discharge**			
Median	30	30	0.792
Interquartile range	20–45	15–45	
**Length of hospital stay (days)**			
Mean (± SD)	6.8 (±5.0)	10.3 (±8.4)	0.006
Range	0–27	1–45	
**Discharge destination, % (N)**			0.51
Previous residence	29.8 (28)	21.2 (7)	
Relative's residence	4.3 (4)	0	
Orthopaedic rehabilitation	55.3 (52)	72.7 (24)	
Care of the elderly ward	3.2 (3)	3.0 (1)	
Other medical specialty ward	2.1 (2)	0	

Two (1.6%) participants voluntarily withdrew mid-study, but all others completed the study (n = 125). Data completion was 86% overall as daily clinical examination was not always appropriate or possible (e.g. patient declining on a given day or in critical condition). Although all volunteers had at least one serum sodium and at least 24 hours of fluid balance measurements, serum sodium measurements were available in 44% of the total patient-days in hospital. Complete fluid balance charts were available for 50% of the total days spent in hospital by participants. Medication data were complete for all cases. None of the hyponatremic participants had urine sodium concentration measured.

### Epidemiology of hyponatremia

Prevalence of hyponatremia upon admission in all identified EPFF was 14.2%. In recruited volunteers, the prevalence of known hyponatremia prior to their fracture was 10.2%. Point prevalence at admission in the recruited group was 13.4%. There were 16 incident cases of hyponatremia developing in hospital (12.6%). Of these incident cases, four (25%) developed pre-operatively, eleven (68.3%) post operatively (one patient died), and one (6.3%) in a patient who was deemed unfit for surgery. The prevalence of known hyponatremia at discharge was 19%. Thus, hyponatremia was detected in 33 participants (26%) and mild hyponatremia (Na 130–134 mmol/L) was predominant (N = 25, 75.8%).

### Etiology of hyponatremia

Hypovolemic hyponatremia was present in 23 cases (69.7%), 9 cases were euvolemic (27.3%) and one case hypervolemic (3%). Etiology was multifactorial in 24/33 cases (72.7%)e. The identified underlying causes and contributory factors are displayed in Table 4. There were no significant differences in causes of hyponatremia between those developing pre- or post-admission.

**Table 4:** Etiologies of case of hyponatremia.

Etiology	% of cases associated (N)
Bendroflumethiazide	75.8 (25)
Dehydration	69.7 (23)
Proton pump inhibitors	69.7 (23)
Syndrome of inappropriate antidiuretic hormone	27.3 (9)
Mirtazapine	15.2 (5)
Glucocorticoid deficiency	6.1 (2)
Reset osmostat	6.1 (2)
Liver cirrhosis	6.1 (2)
Heart failure	6.1 (2)
Furosemide	6.1 (2)
Sertraline	3.0 (1)
Fluid overload	3.0 (1)
Butetamide	3.0 (1)
Olanzapine	3.0 (1)
Amitriptyline	3.0 (1)
Chronic kidney disease	3.0 (1)
Non-steroidal anti-inflammatory drugs	3.0 (1)
Phenobarbitone	3.0 (1)

## Discussion

The findings demonstrate that hyponatremia in EPFF is highly prevalent, occurring in 33/127 cases (26%). Although it had previously been assumed that euvolemic hyponatremia is the commonest type, this study shows that hypovolemic hyponatremia is predominant in EPFF.

The 13.4% point prevalence of hyponatremia upon hospital admission, is within the range of recently reported figures for EPFF, 2.8–26.5% [5], [8], [9], [24]–[26]. Our result is almost identical to that of Gankam Kengne *et al.* who report 13.1% prevalence in EPFF, studying a population with similar proportions of hip and femoral fractures (55%) in a larger scale retrospective study [5]. The 10.2% prevalence of hyponatremia prior to fracture in this study is higher than the 8% prevalence reported in ambulatory patients >60 years and 5.7% in very old non-hospitalised individuals [4], [27]. This may reflect the underlying frailty of those that go on to sustain fragility fractures but is also consistent with the theory that some fractures may be attributable to hyponatremia [7], [16]. The 12.6% incidence of hyponatremia developing in hospital reported here is significantly higher than previous accounts in EPFF, which range from 2.6 to 5.5% [3], [8], [24], [28]. However, unlike previous authors, we reported cases of hyponatremia developing at any stage in hospital rather than solely in the post-operative period. The high incidence of new hyponatremia has important implications for patient welfare as patients who develop hyponatremia in hospital reportedly fare worse than those who are hyponatremic on admission [6], [29], [30]. The prevalence of hyponatremia at discharge was 19%. There are few data in the current literature to compare our figure with. However, it appears common practice not to treat hyponatremia, particularly when mild and chronic, so the prevalence of hyponatremia at discharge is presumably high [5], [31], [32]. This implies that clinician awareness of the potential dangers of hyponatremia is low. Gankam Kengne *et al.* reported that four of eighteen EPFF discharged with hyponatremia had recurrent falls and fractures [5]. In common with comparable studies, most cases of hyponatremia in this study were mild and therefore at risk of being unidentified or ignored [16], [25], [33].

Regarding etiology, hyponatremia was multi-factorial in 72.7% of cases in this study. This is consistent with previous reports that 51.3% and 75.3% of hyponatremia in elderly patients is multifactorial, though both reports concerned severe hyponatremia [18], [19]. However, our findings contrast with previous studies that report that SIADH is the commonest cause of hyponatremia in other patient settings [18], [19]. SIADH was associated with 27.3% of cases of hyponatremia in this study. In a most similar cohort of EPFF, 37% of cases of hyponatremia were attributed to SIADH on discharge diagnosis, all of which were idiopathic [5]. Although SIADH may have no obvious underlying cause, this raises the possibility that SIADH was over-diagnosed [21]. The findings of our study suggest that SIADH is not the commonest cause of hyponatremia in EPFF and that causes related to hypovolemia are more frequent.

Thiazide diuretic therapy (bendroflumethiazide in all instances) was a causative factor in 75.8% of cases of hyponatremia. The high prevalence is greater than that reported in any other series, but is consistent with recent findings that thiazide diuretic therapy is an increasingly important cause [34]–[35]. Gankam Kengne *et al.* reported diuretics were associated with 35% of cases of hyponatremia in EPFF [5]. Hoorn *et al.* observed a higher prescription frequency in hyponatremic EPFF compared to normonatremic controls [33]. In our study, bendroflumethiazide therapy was three times more commonly prescribed in hyponatremic EPFF (21.2%) than in normonatremic EPFF (7.4%) (p = 0.049). This is an important finding given the high prevalence of hypertension in elderly people and the widespread prescribing of thiazide diuretics [34], [35].

Dehydration was associated with 69.7% of hyponatremia, which is in keeping with our previous proposition that many hyponatremic older individuals have appropriate ADH secretion and are, in fact, inadequately hydrated [21]. Dehydration was the result of over-use or incorrect use of diuretics, or inadequate fluid intake or replacement, or a combination of these. Although inadequate fluid and electrolyte management in elderly surgical patients is not a new concern, the prevalence and importance of dehydration in hyponatremia and EPFF is relatively unreported. Absence of reports may stem from the frequent retrospective methodology approaches, reliant on clinical documentation which often lack any record of hydrational state [32]. Given the high prevalence of co-morbidities and polypharmacy in EPFF, cases of hyponatremia caused by dehydration may be misattributed to other causes. Thus dehydration, arguably the simplest and most readily treatable etiology of hyponatremia, could be frequently over-looked and underdiagnosed. This study shows it is a major cause of hyponatremia in EPFF.

Proton pump inhibitor (PPI) therapy (omeprazole in all cases in our study) was associated with 69.7% of cases of hyponatremia. To our knowledge we are the first group to present this finding in EPFF. Reports of omeprazole induced hyponatremia are infrequent and our finding may simply reflect the very high prevalence of PPI prescribing in older hospitalised patients [36]. The prevalence of PPI therapy among hyponatremic patients was 42.4% compared to 35.1% in normonatremic patients (p = 0.51). Another possibility is that few cases of hyponatremia are solely attributable to PPIs but hyponatremia in EPFF is predominantly multifactorial [37]. Rosholm *et al.* report a lower mean serum sodium in very old non-hospitalised individuals prescribed omeprazole compared to controls and emphasise the role of degenerative physiology in adverse drug reactions [27]. Considering the likelihood of degenerative physiology in EPFF and the physiological stress in the post-fracture and peri-operative period, our finding that omeprazole may be a significant contributor to hyponatremia in EPFF is an important one that merits further study.

Antidepressants were commonly associated with hyponatremia in our study. The commonest in our cohort (15.2% of cases) was mirtazapine. Sertraline, and amitryptiline were both associated with a single case each (3%). Our findings are in line with those of other studies. Studying large bone fractures, Sandu *et al.* report that 24.2% of hyponatremic patients were prescribed antidepressants (75% selective serotonin reuptake inhibitors (SSRIs) and 25% mirtazapine), compared to no antidepressant prescriptions in a control group with no fracture [25]. Studying EPFF, Gankam Kengne *et al.* similarly report that 17% of cases of hyponatremia were attributable to SSRIs. Previous studies present conflicting evidence between specific antidepressants and the risk of hyponatremia, and this may simply reflect differences in prescribing patterns [38], [39]. Movig *et al.* report that, in elderly people, concomitant diuretic and SSRI therapy incurs a 13.5 increased odds ratio of developing hyponatremia compared to SSRI therapy alone [39]. The frequent concomitant prescribing of both these drug classes in elderly patients makes this an important observation, especially considering the results of our study.

The remaining etiologies encountered in this study were not surprising and were representative of a general multi-morbid elderly patient cohort. Two independent groups reported that all elderly orthopedic patients becoming hyponatremic post-operatively received hypotonic fluids, mostly in the form of dextrose [3], [40]. We did not find this in our study as hypotonic study was hardly ever prescribed (only to two patients that developed hyponatremia and no more than 1000 mls each in total). However, fluid overload was associated with one case of hyponatremia where the patient had cardiac failure (3%). This may reflect different practices in different centres or may reflect changes in practice with time.

We did not set out to measure outcomes in this study, but the mean length of time from admission to surgery was higher in hyponatremic participants (3 days) compared to normonatremic participants (1.8 days), a 66.7% increase (p = 0.014). This delay is a potential risk factor for development of hyponatremia and suggests patients with admission or pre-operative hyponatremia may have more complicated co-morbidities or fractures and therefore require a pre-operative preparatory period. It also suggests that a delayed time to surgery increases the risk of hyponatremia, potentially by dehydration or inappropriate fluid and electrolyte management. Hyponatremic participants had a 3.5 day longer mean length of hospital stay, compared to normonatremic participants, a 51.5% increase (p = 0.006). Delayed discharge in hyponatremic patients may be caused by attempts to correct the hyponatremia in hospital but it is clear from our study that this is not the case as asymptomatic hyponatremia was usually left untreated. More plausible alternative explanations are that the hyponatremia is a marker of dyshomeostasis or comorbidities which delay discharge, or directly causes subtle cognitive or functional impairments that complicate discharge.

Our study has some important limitations. The observational design did not permit the investigating team to request clinical tests. This was particularly relevant because the clinical management of hyponatremia was frequently sub-optimal and important clinical investigations were not always carried out. Therefore data were missing in some instances.

In these instances the expert panel relied on avaliable clinical information and the over-all clinical picture of each case as it transpired *i.e.* changes in serum sodium compared to iatrogenic interventions. This same limitation has previously been encountered by various other groups [5], [31], [32]. Since patients did not have daily serum sodium measurements, it is likely that our report may underestimate the true incidence of hyponatremia in EPFF. The same issue also complicated the already challenging task of diagnosing etiology of hyponatremia in elderly patients. Although expert panel consensus decision represents the best available method to elucidate causes, there remains considerable uncertainty with regard to diagnosis, especially where information is limited. This is particularly pertinent in relation to diagnosing SIADH, which strictly requires urine sodium concentration measurement and other investigations that are frequently missed [41]. Whilst these are limitations of this study they reflect the reality of inadequate awareness and management of hyponatremia in clinical practice, predisposing these already frail patients to worsened clinical outcomes.

A second limitation is the relatively small sample size of this single centred study meaning that the results may be subject to selection and sampling bias. Moreover adults with incapacity, who compose a significant proportion of EPFF, were excluded from this study as dictated by the ethics committee. Given the independent association between hyponatremia and age, excluding this frail patient cohort at highest risk of hyponatremia (mean age 7 years older than participants), compromises the generalisability of our findings [2]. Furthermore, considering the 12.1% prevalence of hyponatremia among the adults with incapacity identified in this study and the association between hyponatremia and cognitive impairment, including this patient subset in future research ought to be routine. Indeed, hyponatremia may be the precipitant cause of incapacity and so these frailer patients may arguably benefit most from improved understanding of etiology of hyponatremia in EPFF. A third limitation is the possibility of ascertainment bias as individual members of the expert panel may have their own preconceptions on common and uncommon causes. However, discussion amongst the three experts and the use of the diagnostic algorithm served to minimise ascertainment bias, if any was present.

Despite these limitations this study makes a significant contribution to the current understanding of hyponatremia in EPFF. The prospective study design is an improvement over published retrospective comparators. Unlike other published series that typically rely on diagnoses made by clinicians inexpert in hyponatremia, or on retrospective analysis of databases using discharge codes, the present study collected data prospectively that were then reviewed by an expert panel. The attention paid to data regarding volemic state in our study far surpasses previous work. Despite the aforementioned limitations, the involvement of two consultant geriatricians with special interest in hyponatremia, makes this report the most accurate account of etiology of hyponatremia in EPFF to date. The expertise of these individuals not only provided quality assurance of etiology reported for these complex patients, but enabled identification of the likeliest diagnoses in cases lacking all data.

In summary, hyponatremia is highly prevalent in EPFF. This study shows that etiology of hyponatremia in EPFF is frequently multifactorial, mostly drug induced and associated with inadequate fluid replacement therapy. We found SIADH was associated with under a third of cases. This suggests that prevalence and incidence of hyponatremia in EPFF could potentially be reduced by appropriate fluid replacement therapy and careful prescribing. It would be important to confirm this in a further larger, multi-centre study. Although it remains unclear if targeting hyponatremia itself would improve patient outcomes, such interventions have great potential to decrease falls and fractures in older people and generally improve prognosis in EPFF.

## References

[1] Soiza RL, Hoyle GE, Chua MPW (2008) Electrolyte and salt disturbances in older people: causes, management and implications. Rev Clin Gerontol 18: 143–158.

[2] Soiza RL, Talbot HSC (2011) Management of hyponatremia in older people: old threats and new opportunities. Ther Adv Drug Safety 2: 9–17. 10.1177/2042098610394233 PMC4110800 25083198

[3] Tambe AA, Hill R, Livesley PJ (2003) Post-operative hyponatremia in orthopaedic injury. Injury 34: 253–255. 12667775 10.1016/s0020-1383(02)00256-5

[4] Upadhyay A, Jaber BL, Madias NE (2006) Incidence and Prevalence of Hyponatremia. Am J Med 119: S30–S35. 16843082 10.1016/j.amjmed.2006.05.005

[5] Gankam Kengne F, Andres C, Sattar L, Melot C, Decaux G (2008) Mild hyponatremia and risk of fracture in the ambulatory elderly. QJM 101: 583–588. 18477645 10.1093/qjmed/hcn061

[6] Chua M, Hoyle GE, Soiza RL (2007) Prognostic implications of hyponatremia in elderly hospitalized patients. Arch Gerontol Geriatr 45: 253–258. 17244514 10.1016/j.archger.2006.11.002

[7] Ayus JC, Arieff AI (1999) Chronic hyponatremic encephalopathy in postmenopausal women: association of therapies with morbidity and mortality. JAMA 281: 2299–2304. 10386554 10.1001/jama.281.24.2299

[8] Beloosesky Y, Hershkovitz A, Solovey B, Salai M, Weiss A (2011) Hip fracture post-operation dysnatremia and Na+-courses in different cognitive and functional patient groups. Arch Gerontol Geriatr 53: 179–182. 21067828 10.1016/j.archger.2010.10.014

[9] Tolouian R, Alhamad T, Farazmand M, Mulla ZD (2012) The correlation of hip fracture and hyponatremia in the elderly. J Nephrol 25: 789–793. 22135036 10.5301/jn.5000064

[10] Ayus JC, Krothapalli RK, Arieff AI (1987) Treatment of symptomatic hyponatremia and its relation to brain damage. N Engl J Med 317: 1190–1195. 3309659 10.1056/NEJM198711053171905

[11] Ayus JC, Varon J, Arieff AI (2000) Hyponatremia, cerebral edema, and noncardiogenic pulmonary edema in marathan runners. Ann Intern Med 132: 711–714. 10787364 10.7326/0003-4819-132-9-200005020-00005

[12] Ayus JC, Moritz ML (2010) Bone disease as a new complication of hyponatremia: moving beyond brain injury. Clin J Am Soc Nephrol 5: 167–168. 20089487 10.2215/CJN.09281209

[13] Ayus JC, Achinger SG, Arieff A (2008) Brain cell volume in hyponatremia: role of sex, age, vasopressin, and hypoxia. Am J Physiol Renal Physiol 295: F619–F624. 18448591 10.1152/ajprenal.00502.2007

[14] Boscoe A, Paramore C, Verbalis JG (2006) Cost of illness of hyponatremia in the United States. Cost Eff Resour Alloc 4: 10. 16737547 10.1186/1478-7547-4-10 PMC1525202

[15] Barsony J, Manigrasso MB, Xu Q, Tam H, Verbalis JG (2013) Chronic hyponatremia exacerbates multiple manifestations of senescence in male rats. Age 35: 271–288. 22218780 10.1007/s11357-011-9347-9 PMC3592950

[16] Verbalis JG, Barsony J, Sugimura Y, Tian Y, Adams DJ, et al (2010) Hyponatremia-induced osteoporosis. J Bone Mineral Res 25: 554–563. 10.1359/jbmr.090827 PMC3153395 19751154

[17] Renneboog B, Musch W, Vandemergel X, Manto MU, Decaux G (2006) Mild Chronic Hyponatremia Is Associated With Falls, Unsteadiness, and Attention Deficits. Am J Med 119: 71.e1–71.e8. 10.1016/j.amjmed.2005.09.026 16431193

[18] Shapiro DS, Sonnenblick M, Galperin I, Melkonyan L, Munter G (2010) Severe hyponatremia in elderly hospitalized patients: Prevalence, aetiology and outcome. Intern Med J 40: 574–580. 20298512 10.1111/j.1445-5994.2010.02217.x

[19] Clayton JA, Le Jeune IR, Hall IP (2006) Severe hyponatremia in medical in-patients: aetiology, assessment and outcome. QJM 99: 505–511. 16861720 10.1093/qjmed/hcl071

[20] Ayus JC, Negri AL, Kalantar-Zadch K, Moritz ML (2012) Is chronic hyponatremia a novel risk fsctor for hip fracture in the elderly? Nephrol Dial Transplant 27: 3275–3731. 10.1093/ndt/gfs412 PMC3484731 23114899

[21] Ayus JC, Wheeler JM, Arieff AI (1992) Postoperative hyponatraemic encephalopathy in menstruant women. Ann Intern Med 117: 891–897. 1443949 10.7326/0003-4819-117-11-891

[22] Hoyle GE, Chua M, Soiza RL (2011) Volaemic assessment of the elderly hyponatremic patient: reliability of clinical assessment and validation of bioelectrical impedance analysis. QJM 104: 35–39. 20823196 10.1093/qjmed/hcq157

[23] Soiza RL, Hoyle GE (2011) Syndrome of appropriate antidiuretic hormone: difficulties with diagnosing syndrome of inappropriate antidiuretic hormone in older people. Intern Med J 41: 295. 21426472 10.1111/j.1445-5994.2010.02388.x

[24] McPherson E, Dunsmuir RA (2002) Hyponatremia in hip fracture patients. Scott Med J 47: 115–116. 12469566 10.1177/003693300204700506

[25] Sandhu HS, Gilles E, DeVita MV, Panagopoulos G, Michelis MF (2009) Hyponatremia associated with large-bone fracture in elderly patients. Int Urol Nephrol 41: 733–737. 19472069 10.1007/s11255-009-9585-2

[26] Kinsella S, Moran S, Sullivan MO, Molloy MGM, Eustace JA (2010) Hyponatremia Independent of Osteoporosis is Associated with Fracture Occurrence. Clin J Am Soc Nephrol 5: 275–280. 20056759 10.2215/CJN.06120809 PMC2827601

[27] Rosholm J, Nybo H, Andersen Ranberg K, Himmelstrup B, Skjelbo E, et al (2002) Hyponatremia in Very Old Nonhospitalised People: Association with Drug Use. Drugs Aging 19: 685–693. 12381237 10.2165/00002512-200219090-00005

[28] Incalzi RA, Gemma A, Capparella O, Terranova L, Sanguinetti C, et al (1993) Post-operative electrolyte imbalance: Its incidence and prognostic implications for elderly orthopaedic patients. Age Ageing 22: 325–331. 8237621 10.1093/ageing/22.5.325

[29] Gill G, Huda B, Boyd A, Skagen K, Wile D, et al (2006) Characteristics and mortality of severe hyponatremia - a hospital-based study. Clin Endocrinol (Oxf) 65: 246–249. 16886968 10.1111/j.1365-2265.2006.02583.x

[30] Wald RCM, Jaber BL, Price LL, Upadhyay A, Madias NE (2010) Impact of Hospital-Associated Hyponatremia on Selected Outcomes. Arch Intern Med 170: 294–302. 20142578 10.1001/archinternmed.2009.513

[31] Gosch M, Joosten-Gstrein B, Heppner HJ, Lechleitner M (2012) Hyponatremia in geriatric inhospital patients: effects on results of a comprehensive geriatric assessment. Gerontology 58: 430–440. 22722883 10.1159/000339100

[32] Saeed BO, Beaumont D, Handley GH, Weaver JU (2002) Severe hyponatremia: investigation and management in a district general hospital. J Clin Pathol 55: 893–896. 12461050 10.1136/jcp.55.12.893 PMC1769815

[33] Hoorn EJ, Rivadeneira F, van Meurs JB, Ziere G, Stricker BH, et al (2011) Mild hyponatremia as a risk factor for fractures: the Rotterdam Study. J Bone Miner Res 26: 1822–1828. 21381111 10.1002/jbmr.380

[34] Mann SJ (2008) The silent epidemic of thiazide-induced hyponatremia. J Clin Hypertens (Greenwich) 10: 477–484. 18550938 10.1111/j.1751-7176.2008.08126.x PMC8109865

[35] Clayton JA, Rodgers S, Blakey J, Avery A, Hall IP (2006) Thiazide diuretic prescription and electrolyte abnormalities in primary care. Br J Clin Pharmacol 61: 87–95. 16390355 10.1111/j.1365-2125.2005.02531.x PMC1884982

[36] Hamzat H, Sun H, Ford JC, MacLeod J, Soiza RL, et al (2012) Inappropriate Prescribing of Proton Pump Inhibitors in Older Patients Effects of an Educational Strategy. Drugs Aging 29: 681–690. 22775478 10.1007/BF03262283

[37] Durst RY, Pipek R, Levy Y (1994) Hyponatremia caused by omeprazole treatment. Am J Med 97: 400–401. 7942948 10.1016/0002-9343(94)90313-1

[38] Wilkinson TJ, Begg EJ, Winter AC, Sainsbury R (1999) Incidence and risk factors for hyponatremia following treatment with fluoxetine or paroxetine in elderly people. Br J Clin Pharmacol 47: 211–217. 10190657 10.1046/j.1365-2125.1999.00872.x PMC2014168

[39] Movig KLL, Leufkens HGM, Lenderink AW, Van Den Akker VGA, Hodiamont PPG, et al (2002) Association between antidepressant drug use and hyponatremia: a case-control study. Br J Clin Pharmacol 53: 363–369. 11966666 10.1046/j.1365-2125.2002.01550.x PMC1874265

[40] Chung HM, Kluge R, Schrier RW, Anderson RJ (1986) Postoperative Hyponatremia. A Prospective Study. Surv Anesthesiol 30: 292. 3947194

[41] Verbalis JG, Goldsmith SR, Greenberg A, Schrier RW, Sterns RH (2007) Hyponatremia Treatment Guidelines 2007: Expert Panel Recommendations. Am J Med 120: S1–S21. 17981159 10.1016/j.amjmed.2007.09.001

